# Lognormals, power laws and double power laws in the distribution of frequencies of harmonic codewords from classical music

**DOI:** 10.1038/s41598-022-06137-3

**Published:** 2022-02-16

**Authors:** Marc Serra-Peralta, Joan Serrà, Álvaro Corral

**Affiliations:** 1 Centre de Recerca Matemàtica, Edifici C, Campus Bellaterra, 08193 Barcelona, Spain; 2 Departament de Fsica Facultat de Ciències, Universitat Autònoma de Barcelona, 08193 Barcelona, Spain; 3 Dolby Laboratories, 08018 Barcelona, Spain; 4 Departament de Matemàtiques, Facultat de Ciències, Universitat Autònoma de Barcelona, 08193 Barcelona, Spain; 5 Complexity Science Hub Vienna, Josefstädter Straßbe 39, Vienna, 1080 Austria

**Keywords:** Mathematics and computing, Nonlinear phenomena

## Abstract

Zipf’s law is a paradigm describing the importance of different elements in communication systems, especially in linguistics. Despite the complexity of the hierarchical structure of language, music has in some sense an even more complex structure, due to its multidimensional character (melody, harmony, rhythm, timbre, etc.). Thus, the relevance of Zipf’s law in music is still an open question. Using discrete codewords representing harmonic content obtained from a large-scale analysis of classical composers, we show that a nearly universal Zipf-like law holds at a qualitative level. However, in an in-depth quantitative analysis, where we introduce the double power-law distribution as a new player in the classical debate between the superiority of Zipf’s (power) law and that of the lognormal distribution, we conclude not only that universality does not hold, but also that there is not a unique probability distribution that best describes the usage of the different codewords by each composer.

## Introduction

For centuries, physics has dealt with deterministic mathematical laws, such as Newton’s laws of mechanics, the laws of electromagnetism, or the laws of relativity^1^. It were Maxwell and Boltzmann who, in the nineteenth century, discovered probabilistic or statistical laws in the study of the motion of the (hypothetical) particles constituting a gas. The so-famous Planck’s radiation law can be understood as another instance of a probabilistic law. The great insight of Maxwell, Boltzmann, and Planck (and others, like Einstein) was the introduction of probability to infer the mechanics of the constituents of matter and radiation. That insight has been one of the most successful knowledge programs in the history of humankind. Although the just-mentioned examples of probabilistic laws are valid in the “ideal case” (no interaction between the constituents), an interaction (at least with the surroundings) has to be present to ensure the existence of a state of equilibrium. In general, the study of how macroscopic behavior emerges from microscopic interactions (either in equilibrium or out of equilibrium) is the goal of statistical physics.

In ecology, in the social sciences (sociology, economics, demography), and in the study of technological and information networks, one is, in some sense, in a situation similar to statistical physics, in which there is an enormous number of individual entities whose behavior depends on each other, leading to an emerging collective behavior^2,3^. Despite the lack of well-defined underlying microscopic laws, it is remarkable that one may find regular statistical laws describing the aggregated properties of the constituent entities, and even more remarkable that it is the same law which seems to capture a particular but important aspect of many of these systems^4–7^. This ubiquitous and nearly universal law is Zipf’s law^8^, which describes how the constituent entities, or tokens, are distributed into larger groups, or types. In this way, Zipf’s law states that the size distribution of these groups (measured in terms of constituent entities) follows a power-law distribution with a loosely constrained value of the exponent, close to 2 (for the probability mass function). In an additional twist in favor of the meaningfulness of statistical “natural” laws beyond physics, Zipf’s law emerges again when one considers quantitative approaches to human sciences (mainly the study of language^9–13^), where the nature of the interactions between the constituent entities is not so clear^14,15^.

However, there have been serious issues with the Zipf’s paradigm, and with power laws in general, being the most important of them the lack of generality of the results^13,16^ and low statistical rigor^17–22^. In the first case, a very small number of datasets are usually analyzed in order to establish the validity of Zipf’s law in every particular system. For instance, in quantitative linguistics, research articles are usually focused in about a dozen (or even less) texts^23,24^, with the selection of them seeming rather arbitrary. Therefore, many published claims should be considered as anecdotic examples, or conjectures, rather than well-established facts.

Regarding statistical rigor, proper fitting methods and goodness-of-fit tests have seldomly been used, being replaced many times by visual, qualitative checks. Moreover, some apparently rigorous procedures^19^ have been found to yield inconsistent results^25,26^. Therefore, it is not yet established for which systems Zipf’s law is a rough or even bad approximation and for which systems it is a good description in some range or limit. An annoying side effect of the lack of proper statistical tools is the recurrent debate about if the lognormal distribution is superior or not to the power law to describe (some) Zipfian systems^27,28^. A further concern is some ambiguity in the definition of Zipf’s law^13,29,30^, which admits several mathematical formulations not strictly equivalent between them.

In recent years, several authors have tried to overcome the problems of Zipf’s law in linguistics. For instance, Moreno-Sánchez et al.^13^ analyzed different mathematical alternatives to Zipf’s law, using all English texts (tens of thousands) available from the Project Gutenberg digital library. In a sort of complementary study, Mehri and Jamaati^31^ considered just one text (the Bible) but in its translation to one hundred distinct languages. An alternative approach, instead of analyzing many different individual texts, has been to use big corpora (collections such as the British National Corpus formed by gathering many text fragments). Although this is also a valid procedure, one cannot assert that results (for instance, a claimed double power-law distribution comprising Zipf’s law for large word frequencies^32–34^) are not an artifact arising from the mixture of rather different sort of texts^35^. In other words, the statistical properties of the British National Corpus could be different if the corpus were compiled in a different way (e.g., changing the length of the selected fragments), and very different also to the ones of a hypothetical text of the same length from a single author.

In the last years, diverse forms of artistic expressions have been approached through the eyes of complex-systems science^36^. In this paper, we deal with music. Music seems to be a necessary and sufficient condition for “humanity”^37^, in the sense that music has been present across all human societies in all times, and other animal species do not seem to have real musical capabilities. Thus, music is a uniquely human attribute (needless to say, if any extraterrestrial intelligence were ever discovered, one of the first questions to figure out would be about its relationship with some sort of music^38,39^ ). Even more, music is one of the human activities that attracts more public interest (e.g., at the time of writing this, out of the 10 Twitter accounts with more followers, 6 correspond to popular musicians or musical performers^40^ ). Certain parallelisms between natural language and music have been noted in the literature, where music has been sometimes categorized as a “language”^41^; nevertheless, there is no clear notion of grammar and semantic content in music^37,41^ although there exist relations in terms of rhythm, pitch, syntax and meaning^42^.

In any case, one can conceptualize music as a succession (in time) of some musical descriptors or symbols, which can be counted in a Zipf-like manner^43^, with the frequency of appearance of each different symbol playing the role of the size of the groups (types) in which Zipfian systems are organized. A remarkable problem is that, in contrast to language^24^, the individual entities to analyze in music can be extraordinarily elusive to establish. For instance, Manaris et al.^44^ mention several possibilities involving different combinations of pitch and duration, as well as pitch differences, illustrating the multidimensional character of music. This, together with some technicalities to deal with musical datasets, may explain the fact that the study of Zipf’s law and other linguistic laws in music has been substantially limited in comparison to natural language. Nevertheless, some precedents are of interest. Using a simple pitch-duration pair as a metric, Zanette^41^ fitted a variant of Zipf’s law to four classical musical pieces, finding a rather high power-law exponent (up to 4.6 for the probability mass function of frequency, except for a piece by Arnold Schönberg). Later, Liu et al.^45^ did the statistics of pitch jumps for five classical composers to find even higher power-law exponents. In a more large-scale study, Beltrán del Río et al.^46^ analyzed plain pitches (of which there is a maximum “vocabulary” of 128) in more than 1800 MIDI files, containing classical music, jazz, and rock, to fit a generalization of Zipf’s law. This, in general, resulted in a rather high power-law exponent. In any case, these publications did not use very high statistical standards.

In a more recent attempt, studying popular Western music, Serrà et al.^47^ considered the combination of pitch classes present in short time intervals (harmonic and melodic chords, in some sense) to construct discretized chromas. Aggregating individual pieces for fixed lustrums of the twentieth century, and using maximum-likelihood estimation with the necessary goodness-of-fit tests, they found a robust exponent for the tail close to 2.2 (in agreement with Zipf’s law), which remained stable across the different historical periods that were analyzed. Distributions of timbral indicators were also explored in that work, as well as by Haro et al.^48^; intriguingly, the latter reference found that Zipf’s law for timbre is not only fulfilled by music but also by speech and natural sounds (such as rain, wind, and fire).

In this paper, we analyze classical music using its “crystallization” into electronic MIDI scores, by means of a rather large database. One could argue that, in order to access genuine expressions of music, audio recordings are preferable to MIDI scores, due to the fact that the latter may lack the richness and nuances of interpretation^49^ (although there are MIDI files created from the life performance of a musical piece). Nevertheless, for our purposes, scores contain the essence of music, and, in the case of music previous to the twentieth century, they are our best remainder of the original intention of the composer. Moreover, as to undertake statistical analysis we need to deal with discretized elements, scores provide an objective first step in such discretization.

In the next sections, we present the characteristics of the corpus used (“Data, processing, and elementary statistics”), describe the extraction of harmonic codewords from the MIDI files (“Data, processing, and elementary statistics” and Supplementary Information, SI), introduce the probability distributions to fit the codewords counts (power law, double power law, and lognormal, “Power-law, double power-law and lognormal fits”), explain the statistical procedure (“Power-law, double power-law and lognormal fits” and SI), and present the results (“Results”). Naturally, we end with some conclusions. The code used in this paper is available in Github^50^.

## Data, processing, and elementary statistics

### Data

As a corpus of classical music scores we use the *Kunstderfuge* database^51^, including 17,419 MIDI scores of composers from the twelfth to the twentieth century. Different aspects of this musical corpus have been analyzed elsewhere^52,53^. We perform a preliminary cleaning in which we identify and remove traditional songs, anthems, anonymous pieces, and also MIDIs arising from live performances, leading to a remainder of 10,523 files. In general, these files contain the name of the composer and an indication of the name of the piece. Further removing files that we are not able to process, files for which we cannot obtain the bar (and cannot determine therefore the temporal unit), files corresponding to very short pieces, and files corresponding to repeated pieces, we retain 9327 of them corresponding to 76 composers, ranging from Guillaume Dufay (1397–1474) to Olivier Messiaen (1908–1992). The complete list of composers, in chronological order, is provided in Table 1. Details of the data cleaning and the method of detection of repeated pieces in the corpus are provided in the Supplementary Information.

**Table 1:** Name of the 76 classical composers in the *Kunstderfuge* corpus analyzed in this paper.

G. Dufay:	**ln**, dpl;	J. Desprez:	**ln**, dpl;	C. de Morales:	**dpl**, ln;	G. P. da Palestrina:	**ln**, dpl
O. Lassus:	**ln**, dpl;	T. L. de Victoria:	**dpl**, ln;	W. Byrd:	**dpl**, ln;	C. Gesualdo:	**dpl**, ln
J. Dowland:	**dpl**, ln;	C. Monteverdi:	**ln**, pl;	G. Frescobaldi:	**dpl**, ln;	S. Scheidt:	**ln**, pl
J. J. Froberger:	**dpl**, ln;	J. B. Lully:	**dpl**, ln;	J.-H. d'Anglebert:	**ln**, dpl;	D. Buxtehude:	**dpl**, ln
J. Pachelbel:	**ln**;	F. Couperin:	**dpl**;	D. Zipoli:	**ln**, dpl;	A. Vivaldi:	**dpl**, ln
J.-F. Dandrieu:	**dpl**;	T. Albinoni:	**dpl**, ln;	J. S. Bach:	**–**;	D. Scarlatti:	**dpl**, ln
G. F. Händel:	**dpl**;	J.-P. Rameau:	**dpl**, ln;	G. P. Telemann:	**dpl**, ln;	J. Haydn:	**dpl**
J. G. Albrechtsberger:	**ln**, pl;	W. A. Mozart:	**dpl**;	M. Clementi:	**dpl**, ln;	L. van Beethoven:	**dpl**
N. Paganini:	**pl**, ln;	F. Schubert:	**ln**;	J. B. Cramer:	**ln**, pl;	F. Mendelssohn:	**ln**, dpl
F. Chopin:	**dpl**, ln;	R. Schumann:	**dpl**, ln;	H. Berlioz:	**ln**, pl;	F. Liszt:	**ln**, dpl
L. M. Gottschalk:	**ln**, pl;	C.-V. Alkan:	**dpl**;	C. Franck:	**ln**;	G. Bizet:	**ln**, pl
A. Bruckner:	**ln**;	M. Mússorgsky:	**dpl**, ln;	J. Brahms:	**ln**;	P. I. Tchaikovsky:	**ln**, dpl
A. Dvorák:	**ln**, dpl;	A. Guilmant:	**pl**, ln;	E. Grieg:	**ln**, dpl;	C. Saint-Saëns:	**dpl**, ln
I. Albéniz:	**ln**, dpl;	G. U. Fauré:	**ln**;	G. Mahler:	**dpl**, ln;	C. Debussy:	**ln**, dpl
L. Janácek:	**ln**, pl;	S. Joplin:	**pl**, ln;	A. Scriabin:	**ln**, dpl;	M. Reger:	**dpl**, ln
F. Busoni:	**dpl**, ln;	E. Satie:	**ln**, pl;	L. Godowsky:	**dpl**, ln;	S. Karg-Elert:	**pl**, ln
M. Ravel:	**dpl**, ln;	O. Respighi:	**ln**, pl;	S. Rajmáninov:	**ln**, pl;	A. Schoenberg:	**pl**, ln
B. Bartók:	**pl**, ln;	N. Médtner:	**ln**, pl;	G. Gershwin:	**ln**, pl;	S. Prokófiev:	**ln**, pl
Í. Stravinsky:	**pl**, ln;	P. Hindemith:	**ln**, dpl;	D. Shostakóvich:	**dpl**;	O. Messiaen:	**dpl**, ln

The order is chronological (from left to right and from top to bottom, established by the average between birth and death). The results of the best fit (in bold), followed by other good fits are included. pl $=$ (simple) power law, dpl $=$ double power law, ln $=$ lognormal. Number of composers fitted by a unique distribution: 14 (**pl** 0; **dpl** 8; **ln** 6). Number of composers with two good fits: 61 (**pl**, ln 7; **dpl**, ln 25; **ln**, pl 14; **ln**, dpl 15).

### Harmonic codewords

**Figure 1:**
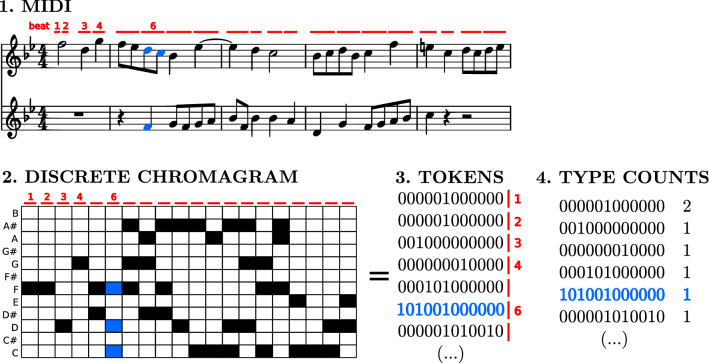
Scheme showing the correspondence between a score (represented by two parallel staves) and its representation in terms of discretized chromas. Counts for each type (number of tokens) are also shown.

Our analysis focuses on the harmonic content of music, understood as the combination of pitches across all instruments in short time frames. A complete summary for the obtention of the elementary units in which we decompose music is provided in the Supplementary Information (see also Ref.^53^); Fig. 1 provides a simple illustration. The different steps are:

(i) Conversion of each MIDI file (corresponding to a piece) into a text file (containing the time occurrence, duration, and pitch of each note).
(ii) Transformation of pitches into (twelve) pitch classes (i.e., collapse into a unique octave).
(iii) Segmentation into elementary time intervals (given by the score beats).
(iv) Construction of chromas: 12-dimensional vectors $(C,C\#,\dots G\#, A,A\#,B)$, counting the contribution of notes from each pitch class for each elementary time interval and for each stave. That is, we collapse all staves in the piece into a single chroma sequence.
(v) Discretization of chromas (using a discretization threshold), which yields the harmonic codewords to analyze. These are 12-dimensional vectors of binary elements (0, 1).
(vi) Transposition to C major (major pieces) or A minor (minor pieces).
(vii) For each composer, aggregation of all the time series of transposed discretized chromas (corresponding to each piece) into a unique dataset.

This aggregation of pieces of the same composer is done in order to get significant statistics. Although under the framework of some models explaining Zipf’s law^54^, aggregation makes little sense, for other models there are no such restrictions^55^. In any case our approach is model free. Notice that aggregation can be done in several ways. Ours is equivalent to aggregate the frequencies *n* of each type, but one could also aggregate the counts *f*(*n*) of each frequency *n*, and this could be done also for relative frequencies. The latter two options would change the results, as they lead to mixtures of the distributions corresponding to each piece.

The obtained codewords contain information about the melody and, mostly, the harmony of the pieces. The most common codewords in the studied corpus are listed in Table 6 of Ref.^53^ and they are consistent with characteristic harmonic features, i.e., they correspond to harmonic sets of pitches. Therefore, the results arising from the analysis of the codewords can be associated with the harmonic characteristics of the compositions, especially in the range of high type frequencies.

### Elementary statistics

For each dataset (corresponding to a composer), we count the repetitions or absolute frequency *n* of each type (discretized chromas, see Fig. 1). This absolute frequency is our random variable, and the number of appearances of each value of the frequency (frequencies of frequencies, then) constitutes an empirical estimation of the probability mass function of the frequency, which we may denote as *f*(*n*). However, due to the broad range of the distributions (with *n* ranging from one to hundreds of thousands), it is more convenient to treat *n* as a continuous random variable and estimate its empirical probability density using logarithmic binning (for visualization purposes only)^20^; so *f*(*n*) denotes in fact a probability density, as well as its empirical estimation.

The number of different types present in a dataset (the types with $n \ge 1$) is what we call the vocabulary of the dataset, denoted by *V* (this is bounded by $2^{12}=4096$). The sum of all the frequencies of all types yields the total number of tokens, which corresponds, by construction, to the dataset length *L* measured in terms of the elementary time unit (number of beats, by default). In a formula, $\sum _{i=1}^V n_i =L$, where *i* labels the types.

## Power-law, double power-law and lognormal fits

### Probability densities and rescaling

**Figure 2:**
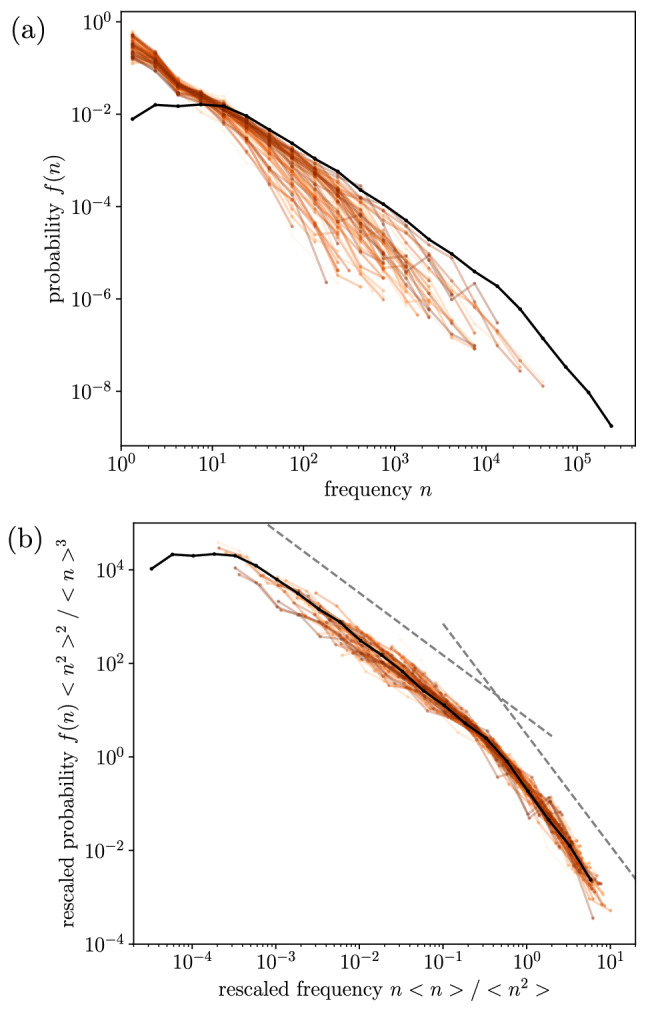
(**a**) Empirical probability densities of codeword frequency for the 76 composers, individually (orange-brown lines), and with all of them aggregated (black line). (**b**) Same distributions under rescaling of the axes, where a roughly similar shape emerges for every composer and the global corpus. Dashed straight lines are power laws with exponents 1.33 and 2.4, derived from the fit of the global dataset.

As a summary of the empirical probability densities of type frequency, Fig. 2a shows all of them (77 in total, one for each composer plus the global one in which all composers are aggregated). All distributions present many types that only occur once ($n=1$, the so-called hapax legomena in linguistics^9^), as well as types with very high frequencies ($n>10^5$ in the global dataset), with a rather smooth decaying curve linking both extremes.

When rescaled, a roughly similar shape is unveiled, as seen in Fig. 2b. Rescaling is done in the following way: $n \rightarrow n \langle n \rangle / \langle n^2 \rangle$ and $f(n) \rightarrow f(n) \langle n^2 \rangle ^2/\langle n \rangle ^3$, with $\langle n\rangle$ and $\langle n^2\rangle$ denoting the first (mean) and second empirical moments of the distribution. The reason behind such rescaling is the assumption of a hypothetical scaling form for *f*(*n*),

$$\begin{aligned} f_\text {sca}(n)=\frac{1}{a} \left( \frac{a}{\theta }\right) ^{\beta _1} G\left( \frac{n}{\theta }\right) , \end{aligned}$$

defined for $n\ge a$, with *a* a (fixed) lower cutoff, $\theta$ a scale parameter, and *G* a scaling function behaving as a decreasing power law with exponent $\beta _1$ for small arguments ($1<\beta _1<2$) and decaying fast enough for large arguments (in order that $\langle n\rangle$ and $\langle n^2 \rangle$ exist). Then, $\langle n \rangle \propto a (\theta /a)^{2-\beta _1}$ and $\langle n^2 \rangle \propto a^2 (\theta /a)^{3-\beta _1}$, and isolating, $\theta \propto \langle n^2 \rangle / \langle n \rangle$ and $\theta ^{\beta _1} \propto a^{\beta _1-1} \langle n^2 \rangle ^2/ \langle n \rangle ^3$, which justifies the rescaling and allows its verification without knowledge of the values of $\theta$ and $\beta _1$, see Ref.^56^. When a literary piece is broken into different parts, this rescaling is equivalent to $n \rightarrow n /L$ and $f(n) \rightarrow f(n) L V$ (see Ref.^57^); however, this is not the case here, and the latter scaling form (in contrast to the former one) does not work well (not shown). Visual inspection of the rescaled plot based on the ratio of moments (Fig. 2b) suggests that the lognormal and the double power law seem appropriate candidate distributions to fit the data.

In this work, we consider three different fitting distributions, all of them continuous; thus, the frequency *n* is assumed to be a continuous random variable. The explanation of the three fitting distributions follows, with special emphasis in the double power law.

### Simple power-law distribution

The simple power-law distribution, referred to as untruncated power law or simply as power-law (pl) distribution, has a probability density

$$\begin{aligned} f_\text {pl}(n)= \frac{\beta -1}{a} \left( \frac{a}{n} \right) ^{\beta } \text{ for } n\ge a, \end{aligned}$$

and zero otherwise. The exponent $\beta$ fulfils $\beta > 1$ and *a* is the lower cut-off, fulfilling $a>0$. Considering *a* as fixed, there is only one free parameter, which is $\beta$. In the particular case in which $\beta$ is in the range $1.8 \le \beta \le 2.2$ we will talk about the fulfillment of Zipf’s law.

### Double power-law distribution

The second fitting distribution is the one we call the double power-law (dpl) distribution^22^, whose probability density is given by

$$\begin{aligned} f_\text {dpl}(n)&= (1-q) \frac{\beta _1-1}{\theta } \frac{1}{c^{1-\beta _1}-1} \left( \frac{\theta }{n} \right) ^{\beta _1} \text{ for } a\le n \le \theta ,\\ f_\text {dpl}(n)&= q \frac{\beta _2-1}{\theta } \left( \frac{\theta }{n} \right) ^{\beta _2} \text{ for } n\ge \theta , \end{aligned}$$

and zero for $n<a$. Naturally, the lower cut-off *a* may take a different value than for the simple power law, although at this point we use the same symbol for simplicity. The two exponents $\beta _1$ and $\beta _2$ fulfill $-\infty< \beta _1 < \infty$ with $\beta _1\ne 1$ and $\beta _2> 1$; $\theta$ is a scale parameter fulfilling $\theta \ge a$; and the lower cut-off *a* fulfills $a \ge 0$ if $\beta _1 < 1$ and $a >0$ if $\beta _1 > 1$. The auxiliary parameter *c* is defined as $c=a/\theta$ and the parameter *q* is not free either but ensures continuity at $n=\theta$ between the two regimes, leading to

$$\begin{aligned} q=\frac{\beta _1-1}{(\beta _2-1) c ^{1-\beta _1} - (\beta _2-\beta _1)}, \end{aligned}$$

which gives $0 < q \le 1$. As the expressions that multiply $1-q$ and *q* in $f_\text {dpl}(n)$ are normalized in their respective ranges, *q* turns out to be the fraction of probability contained in the range $n\ge \theta$. If *a* is fixed, the free parameters are $\beta _1$, $\beta _2$, and $\theta$. The usual (untruncated) power-law distribution is recovered either in the limits $\theta =a$ (equivalent to $q=1$) or $\beta _1=\beta _2 > 1$.

Note that the double power-law distribution has two contributions: on the left ($n\le \theta$) we have a truncated (from above) power-law distribution, with weight $1-q$; on the right ($n\ge \theta$) we have an untruncated power law, with weight *q*. We will take advantage of this fact to fit the double power law to the empirical data, fitting, separately, a truncated power law in the range $a\le n\le b$ (where we have redefined $\theta$ as *b*), and fitting an untruncated power law in $n\ge a_2$, (redefining $\theta$ as $a_2$)^58^. In each fit, $a_2$ and *b* are fixed and considered different, in general.

The method used for the fit is, in both cases, the one explained in Refs.^20,22^ (see the Supplementary Information). If both fits are accepted in some range (in the sense that they cannot be rejected, with $p{-}$value greater than 0.20) and their ranges overlap (in the sense that the upper cut-off *b* of the truncated power law is above the lower cut-off $a_2$ of the untruncated power law, and both power laws cross each other), the double power-law fit is not rejected (provided $a\le 32$, see below). The resulting value of $\theta$ is given by the value of *n* at which both fits cross, which turns out to be

$$\begin{aligned} \theta =\left[ \frac{q}{1-q} \, \frac{\beta _2-1}{\beta _1-1}\, \left( \left( \frac{a}{b}\right) ^{1-\beta _1}-1\right) \frac{a_2^{\beta _2-1}}{b^{\beta _1-1}} \right] ^{\frac{1}{\beta _2-\beta _1}}. \end{aligned}$$

In the case in which the double power-law fit works well, we expect $a_2\simeq \theta \simeq b$ (but with $a_2\le \theta \le b$). The replacements $b\rightarrow \theta$ and $a_2\rightarrow \theta$ can lead to small changes in the fitted values of $\beta _1$ and $\beta _2$; nevertheless, the good visual performance of the fits allows us to disregard such changes.

Although we could have fitted the power laws in the discrete case^13,30^, we have considered the continuous case instead, in order to compare on equal footing with the (truncated) lognormal distribution defined below, which is continuous. For high enough values of *n*, the distinction between continuous and discrete random variables becomes irrelevant, but not for small values of *n*.

The sudden change of exponent of the double power law at $n=\theta$ may seem “unphysical”, but the distribution works quite well for the number of data we are dealing with (in the last section we discuss an extension of the double power law that avoids this “unphysicality”). The case of interest for us is when $0<\beta _1 < \beta _2$; specifically, when $\beta _2$ is between 1.8 and 2.2, the resulting power-law tail is in correspondence with Zipf’s law; then, we will refer to this particular case of a double power-law distribution as “double Zipf”^32^.

### Truncated lognormal distribution

The third fitting distribution that we deal with is the (lower) truncated lognormal (ln), whose probability density is

$$\begin{aligned} f_\text {ln}(n)= {\sqrt{\frac{2}{\pi }}} \left[ \text{ erfc }\left( \frac{\ln a -\mu }{\sqrt{2} \sigma }\right) \right] ^{-1} \frac{1}{ \sigma n} \exp \left( -\frac{(\ln n-\mu )^2}{2\sigma ^2}\right) \text{ for } n\ge a \end{aligned}$$

and zero otherwise, with $a\ge 0$ and $\mu$ and $\sigma$ the two free parameters (being the mean and the standard deviation of the associated untruncated normal distribution); $\text{ erfc }(y)=\frac{2}{\sqrt{\pi }} \int _y^\infty e^{-x^2} dx$ is the complementary error function. The fitting procedure^22^ proceeds in exactly the same way as for the untruncated power law (with the only difference that the lognormal involves two free parameters, $\mu$ and $\sigma$, when *a* is considered fixed).

### Fitting method and model selection

The random variable to fit is the absolute frequency *n* of the codewords (types); this choice is not obvious in Zipfian systems, see Ref.^30^. The fitting method is the one in Refs.^20,22^, consisting in maximum-likelihood estimation and the Kolmogorov-Smirnov goodness-of-fit test for different values of the lower cut-off *a* (and the upper cutoff *b* for the first regime of the double power law). The selected value of *a* (and *b*) is the one that yields the largest number of types in the fitting range (i.e., the one comprising more realizations of the random variable) among all the fits that lead to a $p-$value larger than 0.20. If the resulting value of *a* is larger than $10^{3/2}\simeq 32$ (corresponding to more than 1.5 orders of magnitude from $n=1$ to *a*), the fit is rejected, otherwise it is “accepted” and considered a “good fit”.

For the comparison of the fits provided by the different distributions, we have to deal with different subsets of the data (as the values of the lower cut-off *a* will be different in each case, in general). We take the simple criterion of selecting the fitting distribution that yields the smaller value of *a*, i.e., the model that explains the larger portion of the data. The result is what we refer to as the “best fit”. This is explained in detail in the Supplementary Information.

## Results

### Simple power-law and double power-law fits

**Figure 3:**
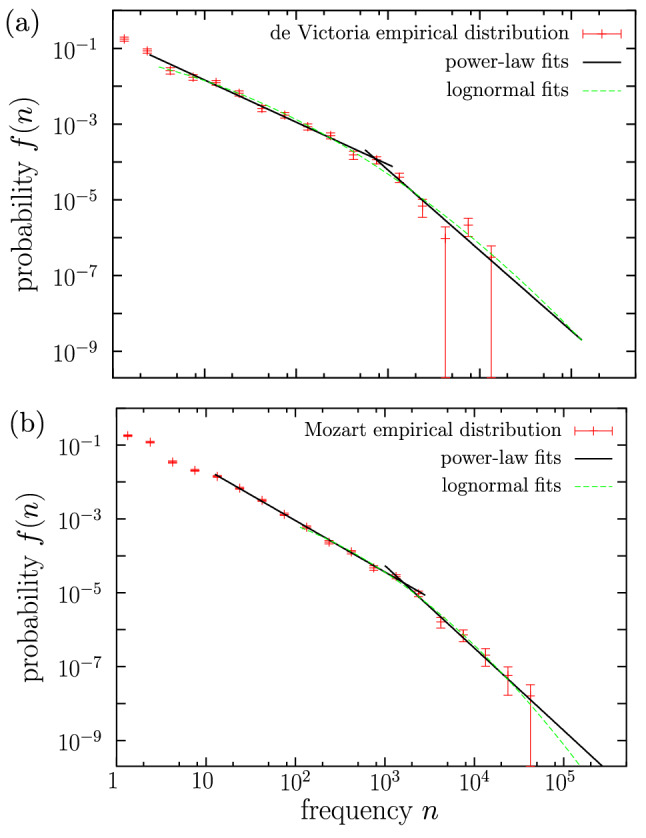
Empirical probability density of codeword frequency for (**a**) de Victoria and (**b**) Mozart, together with double power-law and lognormal fits. Among all the composers, de Victoria and Mozart yield the largest logarithmic span of the fitting range, $n_{max}/a$, for the double power law. Note that de Victoria also yields the lognormal fit with the largest $n_{max}/a$ (comparable to the value for the double power law).

We start by comparing the results of fitting a simple power law and a double power law. We find that for the majority of the composers (48 of them, 63%) the double power law provides a good fit, which is obviously preferable to the simple power law (due to the fact that the double power law covers a larger range of data than the simple power law, as the latter is a part of the former). In fact, when the double power law fits the data, the simple power law is rejected, as this only fits the tail, which is a too small fraction of the data (with $a_\text {pl}\gg 32$). A couple of particularly good double power-law fits are shown as an illustration in Fig. 3, where empirical probability densities and their fits are plotted together; there, it is also clear how the simple power law fits a rather small part of the data ($n>a_\text {pl}\simeq 10^3$).

**Figure 4:**
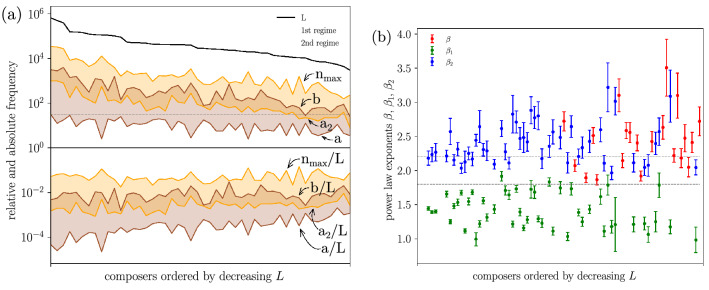
Results of the double power-law fit for each composer for which this is not rejected. (**a**) Fitting ranges sorted by decreasing *L*. Relative fitting ranges, in terms of cut-off frequencies divided by *L*, are also shown below. The dashed line marks the $10^{1.5}\simeq 32$ limit. The middle region not in the legend corresponds to the overlap between both regimes. (**b**) Double power-law exponents $\beta _1$ and $\beta _2$. The value of the exponent $\beta$ for the simple power law (when this is not rejected) is also included. Horizontal dashed lines delimit the Zipf’s range for the exponents $\beta _2$ and $\beta$.

Figure 4 shows, for each composer for which the double power-law fit is not rejected, the corresponding fitting ranges and exponents. We see that when the cut-offs are expressed in terms of the relative frequency, these are much more stable between different composers than when expressed in absolute frequencies (except for the relative minimum cut-off, $a_\text {dpl}/L$). As a rough summary of the figure, the relative scale parameter of the dpl is $\theta /L \approx 0.005$, and the maximum relative frequency is $n_{max}/L \approx 0.05$ (both with considerable dispersion). Curiously, 0.05 is also, approximately, the relative frequency of the most common word in English, which is “the”^59^.

We also see in the figure that the exponent $\beta _1$ ranges between 1 and 2, for most of the composers. This power-law regime coincides qualitatively with what has been found in linguistics, using large corpora (where the exponent $\beta _1$ seems to be between 1.4^33,34^ and 1.6^32,60,61^), but we are not aware that it was reported before in music. In addition, we observe that $\beta _2$ ranges mostly between 2 and 3, (remember that in order to consider that we have a Zipfian tail, $\beta _2$ should be between 1.8 and 2.2, roughly). As the composers are ranked by decreasing *L*, we also observe that the smaller *L*, the larger the dispersion in the values of $\beta _2$ and $\beta$.

However, there are a number of cases (28, 37%) in which the double power-law fit is not appropriate, and this can be due to two main reasons: either the two power-law regimes do not overlap (6 composers) and thus the fit is rejected, or the first power-law regime is meaningless and thus the fit is also rejected. The latter can arise from two subcases: from a too short fitting range (2 composers), or from the fact that both exponents are nearly the same, i.e., $\beta _1\simeq \beta _2$, and then the existence of two power-law regimes cannot be established (20 composers).

**Figure 5:**
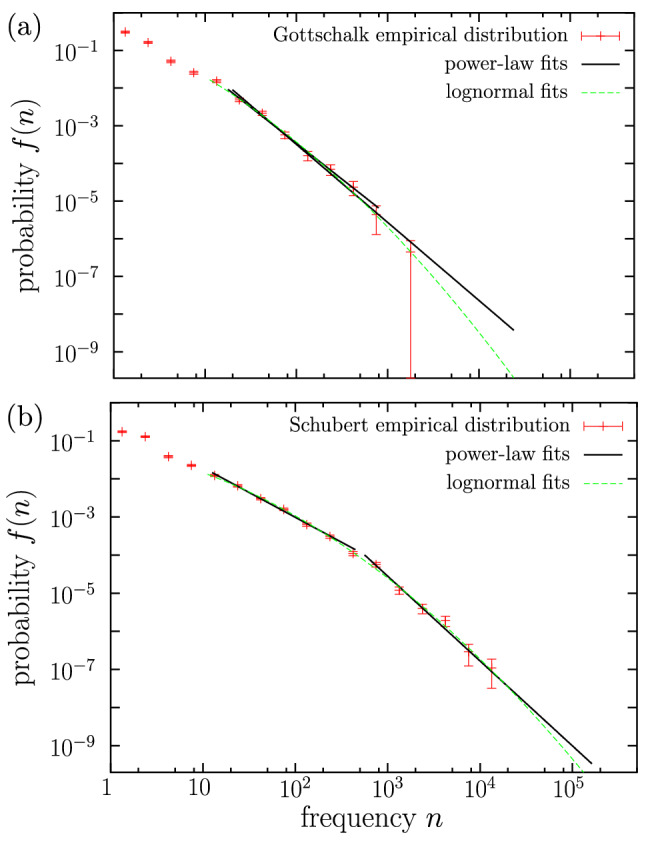
Empirical probability density of codeword frequency for (**a**) Gottschalk and (**b**) Schubert, together with their fits. Gottschalk is the composer with the largest logarithmic fitting range for the simple power law, nevertheless, the (logarithmic) fitting range for the lognormal is larger. Schubert has the largest number of types in the lognormal fitting range (1102). Failed double-power law fits are also shown.

Table 2 displays the results for these two subcases, clearly showing the failure of the double power law ($b/a_\text {dpl}$ small or $\beta _1\simeq \beta _2$; the table makes these statements quantitative). The table also shows that, when the first power-law regime (the truncated one) is meaningless, the simple power law provides a good fit for all composers but one (21 composers; Bruckner, with $a_\text {pl}=35$, is excluded). In most of the cases, the fitted power-law exponent ranges from $\beta \simeq 1.9$ to 2.7 (also displayed at Fig. 4). Figure 5a shows one case of the failure of the double power-law fit and the validity of the simple power law.

**Table 2:** Results of the double power-law fit when this fit is rejected, either because the truncated power-law regime has a too short range or because there are not two different power-law regimes (case of non-overlapping fitting ranges is not included, except for the global dataset)^58^.

Composer	*V*	*a*	*b*	$\beta _1$	$a_2$	$n_\text {max}$	$\ell$	$v_2$	$\beta _2$
**Monteverdi**	247	8	319	1.895 ± 0.126	10	319	1.50	68	**2.048 ± 0.142**
**Scheidt**	233	11	475	2.105 ± 0.155	11	475	1.63	76	**2.184 ± 0.139**
Albrechtsberger	511	16	282	2.634 ± 0.205	16	282	1.25	84	2.632 ± 0.172
**Paganini**	713	10	351	1.638 ± 0.097	8	351	1.65	166	**1.919 ± 0.072**
Cramer	657	11	56	1.786 ± 0.249	20	218	1.04	50	3.098 ± 0.330
**Berlioz**	805	11	398	1.844 ± 0.097	18	602	1.53	109	**2.146 ± 0.104**
**Gottschalk**	1035	18	794	1.908 ± 0.086	20	2410	2.08	174	**2.072 ± 0.084**
**Bizet**	782	11	751	1.709 ± 0.080	13	751	1.78	155	**1.864 ± 0.075**
Bruckner	1602	40	891	2.338 ± 0.157	35	895	1.40	132	2.436 ± 0.119
Guilmant	522	9	276	2.349 ± 0.159	9	276	1.49	92	2.410 ± 0.149
Janáček	874	16	251	2.323 ± 0.148	18	251	1.15	125	2.556 ± 0.147
Joplin	634	8	325	2.154 ± 0.116	8	325	1.61	119	2.220 ± 0.099
**Satie**	912	11	781	1.728 ± 0.073	13	781	1.79	180	**1.894 ± 0.073**
Karg-Elert	752	9	101	2.594 ± 0.225	9	101	1.05	75	2.720 ± 0.210
Respighi	891	16	348	2.304 ± 0.139	16	348	1.34	122	2.404 ± 0.116
Rajmáninov	2231	13	380	2.375 ± 0.099	18	380	1.33	146	2.512 ± 0.117
Schoenberg	997	10	335	2.261 ± 0.212	9	335	1.58	68	2.474 ± 0.200
Bartók	1766	13	226	2.503 ± 0.136	13	226	1.25	153	2.584 ± 0.119
Médtner	1233	13	74	2.825 ± 0.295	16	74	0.67	49	3.504 ± 0.418
Gershwin	1827	16	50	2.398 ± 0.291	22	154	0.84	81	3.100 ± 0.241
Prokófiev	1171	13	291	2.345 ± 0.164	13	291	1.36	93	2.426 ± 0.149
Stravinsky	2442	28	296	2.574 ± 0.159	28	296	1.02	154	2.723 ± 0.141
All 76	4085	40	3980	1.334 ± 0.017	10,000	254,000	1.40	118	2.369 ± 0.120

The simple power law fit is accepted (with $\beta =\beta _2$) for all composers but one (Bruckner, for which $a_\text {pl}=a_2=35$). The number of orders of magnitude of this fit is given by $\ell =\log _{10}(n_\text {max}/a_2)$. The number of types included in the fit is $v_2$. The uncertainties in $\beta _1$ and $\beta _2$ are given by the standard deviation of the maximum likelihood estimation. The rest of variables are explained in the main text. The Zipfian subcases ($1.8 \le \beta \le 2.2$) are marked in bold.

**Figure 6:**
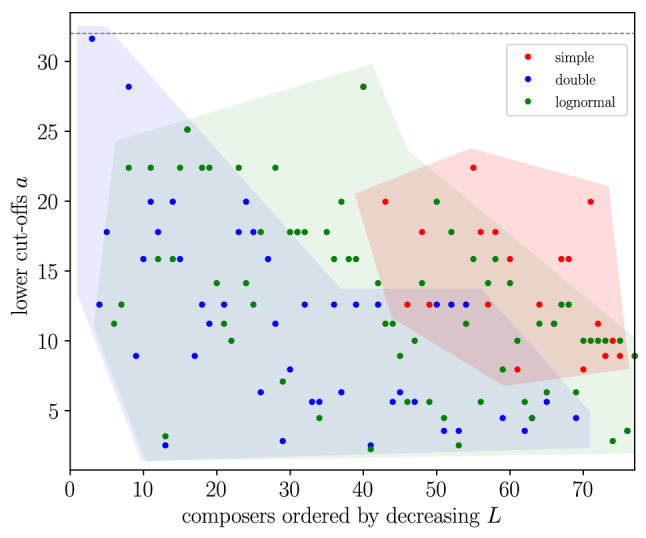
Results for each composer of the lower cut-off for each fit, when this is not rejected. Shadowed regions group the results for each fit. A lower value of *a* implies a larger fitting range; the distribution with the lowest value of *a* is the preferred best fit for each composer.

Coming back to the case when the double power law is rejected because no overlap between the two regimes exists (6 composers only, as Schubert in Fig. 5b), the simple power law is rejected as well, as the value of $a_\text {pl}$ turns out to be too high ($a_\text {pl} \gg 32$) and the power-law fit only includes the tail of the empirical distribution. Nevertheless, we will see that in 5 of these cases the lognormal provides a good fit (see Fig. 5b); the lognormal becomes the best fit then, as represented in Fig. 6. The exception to this is given by Bach, who turns out to be the only composer for which none of the three distributions is able to fit the data with $a\le 32$.

**Figure 7:**
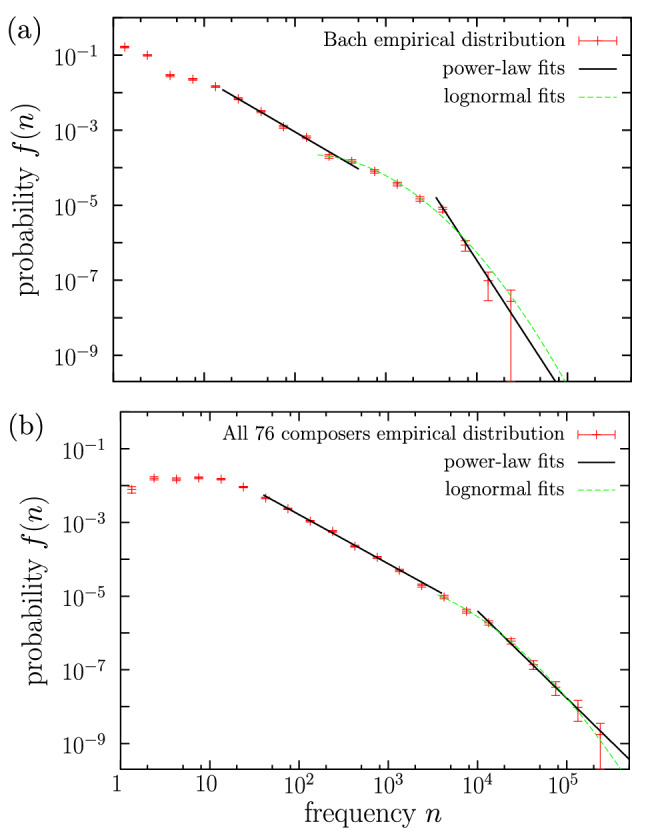
Empirical probability density of codeword frequency for (a) Bach, and for (b) the global dataset with all the 76 composers, together with power-law fits and lognormal fit. In both cases the double-power law fit is rejected, as the two fitted regimes do not overlap, and also the lognormal fit is unsatisfactory, as it excludes a significant part of the types (i.e., the value of *a* turns out to be too large).

We show Bach’s empirical distribution of frequencies together with its different (failed) fits in Fig. 7a; there one can see that the two power-law regimes are far from overlapping, and that the tail exponent is rather large ($\beta \simeq 3.7$) and limited to a narrow range in frequency. Note that Bach’s distribution could be fitted by a power-law body followed (with overlap) by a lognormal tail, but such a distribution is not considered in this paper (of course, other distributions with a supercritical bump could be considered as well^62^).

As another example of a failed double power-law fit, due to the non-overlapping of the two regimes, we consider in Fig. 7b the global dataset of the 76 composers, although it is remarkable that in this case the double power-law fit is not far from being “accepted” (the region of no overlap has a rather short range). The two power law exponents would be $\beta _1=1.33$ and $\beta _2=2.4$ (this case has also been included in Table 2).

### Lognormal fit

Regarding the lognormal fit, it is rejected for only 9 composers (12%), due to a value of $a_\text {ln}$ too large (greater than 32). These are Couperin, Dandrieu, Bach, Händel, Haydn, Mozart, Beethoven, Alkan, and Shostakóvich, for which the double-power law provides a good fit, except for Bach. The values of the cut-offs for the remaining 67 are shown in Fig. 6 (this includes all cases that were not fitted by the double power law, except Bach). When comparing the double power law with the lognormal, we find that in many cases the two distributions provide roughly similar fits (see for instance Fig. 3a; in Fig. 3b, despite some similarity, the fitting ranges are clearly different). Out of the 40 composers that are fitted by both distributions, for 25 of them the double power law provides the best fit (in the sense that it fits a larger fraction of the data), and for 15 of them the situation is reversed (Fig. 6).

All composers fitted by the simple power law (21) are also fitted by the lognormal, and the fit provided by the lognormal is better for 14 of these 21 composers (for the remaining 7 composers the simple power law is able to fit a larger range than the lognormal; this can be seen in Fig. 6). Figure 5a is a good illustration of the case when the lognormal outperforms the simple power law. Figure 5b shows another case, in which both the simple and the double power-law fail but not the lognormal.

## Discussion and conclusions

Table 3 summarizes our results. Recapitulating, the lognormal is the distribution that gives a good fit for more composers (67 out of 76, 88%), followed by the double power law (48, 63%), and by the simple power law (21, 28%). If one considers the simple power law as a special case of the double power law, one would obtain a larger number for the latter distribution (69, 91%), thus accounting for roughly the same percentage of fits as the lognormal. Note that good simple power-law fits arise for composers with low representativity in the corpus (low value of *L* and smaller absolute frequencies); so, we conjecture that increasing the number of pieces by these composers would unveil a range of smaller relative frequencies which could be well fitted by another power-law regime and thus a double power law would emerge in these cases (in other words, for the number of pieces in the corpus of these composers, the value of $\theta$ would be too small to be detectable).

Among the double power-law fits, there are only 15 cases that can be considered good “double Zipf” fits (with $1.8 \le \beta _2 \le 2.2$), and among the simple power-law fits, there are 7 with an exponent in the Zipf range. So, out of 76 composers only 22 (29%) can be considered to follow Zipf’s law in the sense of a good fit (although a lognormal can provide a better fit for some of them). Considering best fits only, Zipf’s law only arises for 9 composers (12%).

**Table 3:** Summary of the fits for the 76 composers.

	Simple pl	Double pl	Lognormal
Best fits	7	33	35
Best Zipf	1	8	–
Good fits	21	48	67
Good Zipf	7	15	–
Reason for rejection	$a_\text {pl}>32$ (55)	No overlap (6)	$a_\text {ln} > 32$ (9)
		$$b \approx a_\text {dpl}$$ (2)	
		$$\beta _1 \approx \beta _2$$ (20)	

First row counts distributions yielding the best fits (in terms of fitting more data); Second row counts the Zipf subcases among the best fits (exponents $\beta$ and $\beta _2$ between 1.8 and 2.2); third counts distributions yielding good fits (fit not rejected but not necessarily the best); and fourth counts the Zipf subcases among the good fits. The statistics for the reason of rejection (bad fits) are also included (in brackets).

**Figure 8:**
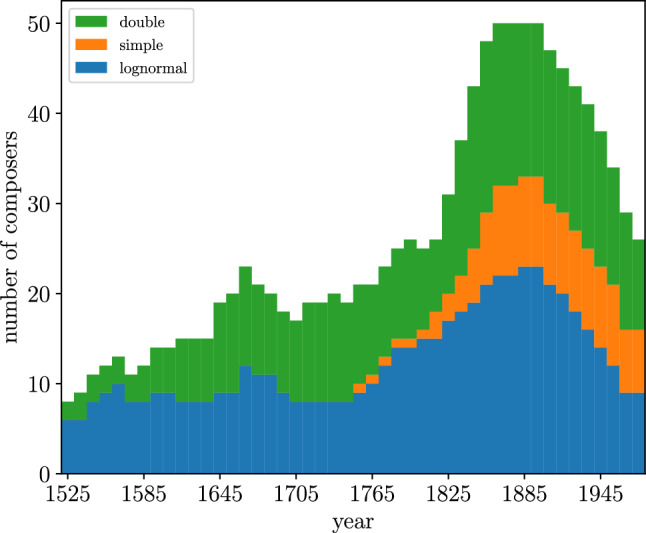
Number of composers best fitted by each fit, as a function of year (year is determined as the mean between birth plus 20 and death). Numbers have been smoothed: for each interval of 10 years we take a window of 150 years and count the number of fits in the window.

If we ask instead, not which distributions fit well the data but for the distribution that yields the best fit (in the sense of fitting more data, remember), Table 3 shows that the lognormal does it for 35 composers, the double power law for 33, and the simple power law for 7; one case (Bach) is not fitted well by any of the three distributions. Figure 8 quantifies these differences in chronological order. Except for the fifteenth century, the double power law slightly dominates about the first half of the data, but after Beethoven, the lognormal takes over. Interestingly, we observe that the simple power law only starts to appear as a best fit in the second part of the corpus, around the eighteenth century, and corresponds to composers with little representation in the corpus (low *L*). Nevertheless, composers with low *L* can also be found in the first part of the corpus, but the simple power law does not provide the best fit in any of such cases.

For a small number of composers (six, as well as for the global aggregated dataset), the double power law is rejected because the two power-law regimes do not overlap. This is an unfortunate situation, as the two power-law regimes exist, but the double power law turns out to be too sharp in its transition from one power-law regime to the other at $n=\theta$. A smoother version of the double power law (sdpl) could be used instead,

$$\begin{aligned} f_\text {sdpl}(n)= \frac{\gamma }{B \theta } \left( \frac{\theta }{n}\right) ^{\beta _1} \left[ \frac{1}{1+\left( n/\theta \right) ^\gamma }\right] ^{\frac{\beta _2-\beta _1}{\gamma }}, \text{ for } n\ge a \end{aligned}$$

where *B* refers to the incomplete beta function

$$\begin{aligned} B\left( \frac{\theta ^\gamma }{\theta ^\gamma + a^\gamma }; \frac{\beta _2-1}{\gamma },\frac{1-\beta _1}{\gamma }\right) \end{aligned}$$

and the extra parameter $\gamma >0$ controls the sharpness of the transition^63^. The limit $\gamma \rightarrow \infty$ recovers the (infinitely sharp) double power law and the limit $a=\beta _1=0$ with $\gamma =1$ leads to the Pareto distribution. In general, one could fix $\gamma =1$ to reduce the number of free parameters, but this seems a rather arbitrary decision. An even more convenient option would be to consider the discrete version of this distribution (as the data are discrete). These extensions are left for future research.

A relevant issue in our analysis is the construction of the harmonic codewords from the scores. The discretization procedure looks for the presence (1) or absence (0) of each pitch class in every beat of the score. This involves two somewhat arbitrary decisions: first, presence and absence are decided in terms of an arbitrary threshold applied to the (nonbinary, continuous) chromas; second, it is taken for granted that the fundamental time unit is the beat. We have tested the robustness of our results against the change in these arbitrary parameters, repeating the process for different discretization thresholds and different selections of the elementary time unit. It is clear that the increase of the time unit (e.g., from one beat to two beats) leads to a reduction in the number of tokens *L* and subsequently in the values of the frequencies *n*. Obviously, scale parameters (such as $\theta$ and $e^\mu$) are strongly affected under such a change; however, a rescaled plot such as the one in Fig. 2b shows that the shape of the distributions is robust and remains nearly the same, also when the threshold is changed. As the distinction between lognormals and power laws depends on the shape and not on the scale of the distributions, our conclusions regarding the lack of universality and the poor fulfilment of Zipf’s law in music do not change (in a previous study utilizing the same discretization method, we also found the definition of the codeword threshold and the temporal unit rather irrelevant given a reasonable range^53^).

In summary, we find that the usage of harmonic vocabulary in classical composers may seem universal-like at a qualitative level (see Fig. 2b), but this paradigm fails when one approaches the issue in a quantitative statistical way. Not only universal parameters to describe the distributions do not exist, but different distributions (lognormal and power laws) fit better different composers. In particular, the Zipf picture (a power-law tail with exponent in the approximate range 1.8–2.2) only applies to a reduced subset of composers (9 best fits, out of 76 composers, and 22 good fits, out of 69 power law and double power-law good fits and 76 composers, see Table 3). Although some degree of universality has been claimed in complex systems in analogy to statistical physics^64^, detailed analyses in some particular systems have shown a diversity of parameters and distributions for some particular systems^22,65^.

Our work can be put into the wider context of quantifying the universality of scaling laws. This is directly related to the use of proper statistical methodologies in complex-systems science, where some controversies have arisen in recent years. For example, the application of the ideas of allometric scaling to urban science^66^ has raised important concerns^67,68^ (see also Ref.^69^ for a revision of the original problem). Methods of fitting power-law distributions have been criticized^19^ and re-criticized^25,26,70,71^. The important role played by statistical dependence when fitting has been pointed out in Ref.^21^. In general, one important lesson that emerges from the study of complex systems is that these have to be characterized in probabilistic terms, so a precise description of their stochastic or probabilistic properties, together with adequate statistical tools, becomes mandatory.

## Supplementary Information

Supplementary Information.

## References

[1] Longair MS (2003). Theoretical Concepts in Physics: An Alternative View of Theoretical Reasoning in Physics.

[2] Watts DJ (2007). A twenty-first century science. Nature.

[3] West G (2017). Scale: The Universal Laws of Life and Death in Organisms, Cities and Companies.

[4] Camacho J, Solé RV (2001). Scaling in ecological size spectra. Europhys. Lett..

[5] Axtell RL (2001). Zipf distribution of U.S. firm sizes. Science.

[6] Adamic LA, Huberman BA (2002). Zipf’s law and the Internet. Glottometrics.

[7] Pueyo S, Jovani R (2006). Comment on “A keystone mutualism drives pattern in a power function”. Science.

[8] Li W (2002). Zipf’s law everywhere. Glottometrics.

[9] Baayen H (2001). Word Frequency Distributions.

[10] Baroni M, Lüdeling A, Kytö M (2009). Distributions in text. Corpus Linguistics: An International Handbook.

[11] Zanette, D. Statistical patterns in written language. arXiv:14123336v1, (2014).

[12] Piantadosi ST (2014). Zipf’s law in natural language: A critical review and future directions. Psychon. Bull. Rev..

[13] Moreno-Sánchez I, Font-Clos F, Corral A (2016). Large-scale analysis of Zipf’s law in English texts. PLoS ONE.

[14] Stephens GJ, Bialek W (2010). Statistical mechanics of letters in words. Phys. Rev. E.

[15] Corral A, García del Muro M (2020). From Boltzmann to Zipf through Shannon and Jaynes. Entropy.

[16] Gerlach M, Font-Clos F (2020). A standardized Project Gutenberg Corpus for statistical analysis of natural language and quantitative linguistics. Entropy.

[17] Bauke H (2007). Parameter estimation for power-law distributions by maximum likelihood methods. Eur. Phys. J..

[18] White EP, Enquist BJ, Green JL (2008). On estimating the exponent of power-law frequency distributions. Ecology.

[19] Clauset A, Shalizi CR, Newman MEJ (2009). Power-law distributions in empirical data. SIAM Rev..

[20] Deluca A, Corral A (2013). Fitting and goodness-of-fit test of non-truncated and truncated power-law distributions. Acta Geophys..

[21] Gerlach M, Altmann EG (2019). Testing statistical laws in complex systems. Phys. Rev. Lett..

[22] Corral A, González A (2019). Power law distributions in geoscience revisited. Earth Space Sci..

[23] Font-Clos F, Boleda G, Corral A (2013). A scaling law beyond Zipf’s law and its relation to Heaps’ law. New J. Phys..

[24] Corral A, Boleda G, Ferrer-i-Cancho R (2015). Zipf’s law for word frequencies: Word forms versus lemmas in long texts. PLoS ONE.

[25] Corral A, Font F, Camacho J (2011). Non-characteristic half-lives in radioactive decay. Phys. Rev. E.

[26] Voitalov I, van der Hoorn P, van der Hofstad R, Krioukov D (2019). Scale-free networks well done. Phys. Rev. Res..

[27] Malevergne Y, Pisarenko V, Sornette D (2011). Testing the Pareto against the lognormal distributions with the uniformly most powerful unbiased test applied to the distribution of cities. Phys. Rev. E.

[28] Corral A, Udina F, Arcaute E (2020). Truncated lognormal distributions and scaling in the size of naturally defined population clusters. Phys. Rev. E.

[29] Mandelbrot B, Jakobson R (1961). On the theory of word frequencies and on related Markovian models of discourse. Structure of Language and its Mathematical Aspects.

[30] Corral A, Serra I, Ferrer-i-Cancho R (2020). Distinct flavors of Zipf’s law and its maximum likelihood fitting: Rank-size and size-distribution representations. Phys. Rev. E.

[31] Mehri A, Jamaati M (2017). Variation of Zipf’s exponent in one hundred live languages: A study of the Holy Bible translations. Phys. Lett. A.

[32] Ferrer i Cancho R, Solé RV (2001). Two regimes in the frequency of words and the origin of complex lexicons: Zipf’s law revisited. J. Quant. Linguist..

[33] Montemurro MA (2001). Beyond the Zipf-Mandelbrot law in quantitative linguistics. Physica A.

[34] Corral A, Serra I (2020). The brevity law as a scaling law, and a possible origin of Zipf’s law for word frequencies. Entropy.

[35] Williams JR, Bagrow JP, Danforth CM, Dodds PS (2015). Text mixing shapes the anatomy of rank-frequency distributions. Phys. Rev. E.

[36] Perc M (2020). Beauty in artistic expressions through the eyes of networks and physics. J. R. Soc. Interface.

[37] Ball P (2010). The Music Instinct.

[38] Jet Propulsion Laboratory, NASA. The golden record. https://voyager.jpl.nasa.gov/golden-record/.

[39] Wikipedia. Voyager golden record. https://en.wikipedia.org/wiki/Voyager_Golden_Record.

[40] Wikipedia. List of most-followed Twitter accounts. https://en.wikipedia.org/wiki/List_of_most-followed_Twitter_accounts.

[41] Zanette DH (2004). Zipf’s law and the creation of musical context. Mus. Sci..

[42] Patel AD (2008). Music, Language, and the Brain.

[43] Zanette D (2008). Playing by numbers. Nature.

[44] Manaris, B., Purewal, T. & McCormick, C. Progress towards recognizing and classifying beautiful music with computers: MIDI-encoded music and the Zipf-Mandelbrot law. In *Proceedings IEEE SoutheastCon 2002 (Cat. No.02CH37283)* 52–57 (2002).

[45] Liu L, Wei J, Zhang H, Xin J, Huang J (2013). A statistical physics view of pitch fluctuations in the classical music from Bach to Chopin: Evidence for scaling. PLoS ONE.

[46] Beltrán del Río M, Cocho G, Naumis GG (2008). Universality in the tail of musical note rank distribution. Physica A.

[47] Serrà J, Corral A, Boguñá M, Haro M, Arcos JLl (2012). Measuring the evolution of contemporary western popular music. Sci. Rep..

[48] Haro M, Serrà J, Herrera P, Corral A (2012). Zipf’s law in short-time timbral codings of speech, music, and environmental sound signals. PLoS ONE.

[49] Hennig H, Fleischmann R, Geisel T (2012). Musical rhythms: The science of being slightly off. Phys. Today.

[50] Chromagramer. https://github.com/MarcSerraPeralta/chromagramer.

[51] kunstderfuge.com. The largest resouce of classical music in .mid files. http://www.kunstderfuge.com.

[52] González-Espinoza A, Martínez-Mekler G, Lacasa L (2020). Arrow of time across five centuries of classical music. Phys. Rev. Res..

[53] Serra-Peralta M, Serrà J, Corral A (2021). Heaps’ law and vocabulary richness in the history of classical music harmony. EPJ Data Sci..

[54] Simon HA (1955). On a class of skew distribution functions. Biometrics.

[55] Cattuto C, Loreto V, Pietronero L (2007). Semiotic dynamics and collaborative tagging. Proc. Natl. Acad. Sci. USA.

[56] Corral A (2015). Scaling in the timing of extreme events. Chaos. Solit. Fract..

[57] Corral A, Font-Clos F (2017). Dependence of exponents on text length versus finite-size scaling for word-frequency distributions. Phys. Rev. E.

[58] In the fitting of the truncated power law, the method we use [20,22] considers the possibility of $$b>n_\text{max}$$ (the truncation point could be above the maximum value of the random variable). for the case we are interested in (the truncated power law giving one of the regimes of the double power law), this does not apply, and therefore we have ignored this fact.

[59] Google Books Ngram Viewer. https://books.google.com/ngrams.

[60] Petersen AM, Tenenbaum JN, Havlin S, Stanley HE, Perc M (2012). Languages cool as they expand: Allometric scaling and the decreasing need for new words. Sci. Rep..

[61] Gerlach M, Altmann EG (2013). Stochastic model for the vocabulary growth in natural languages. Phys. Rev. X.

[62] Corral A, Garcia-Millan R, Moloney NR, Font-Clos F (2018). Phase transition, scaling of moments, and order-parameter distributions in Brownian particles and branching processes with finite-size effects. Phys. Rev. E.

[63] The incomplete gamma function arises immediately with the change of variables $$t=n^\gamma /(\theta ^\gamma +n^\gamma )$$.

[64] Stanley HE (1999). Scaling, universality, and renormalization: Three pillars of modern critical phenomena. Rev. Mod. Phys..

[65] Hantson S, Pueyo S, Chuvieco E (2016). Global fire size distribution: From power law to log-normal. Int. J. Wildl. Fire.

[66] Bettencourt L, West G (2010). A unified theory of urban living. Nature.

[67] Arcaute E (2015). Constructing cities, deconstructing scaling laws. J. R. Soc. Interface.

[68] Leitão JC, Miotto JM, Gerlach M, Altmann EG (2016). Is this scaling nonlinear?. R. Soc. Open Sci..

[69] Ballesteros FJ, Martinez VJ, Luque B, Lacasa L, Valor E, Moya A (2018). On the thermodynamic origin of metabolic scaling. Sci. Rep..

[70] Barabási, A.-L. Love is all you need. Clauset’s fruitless search for scale-free networks. https://www.barabasilab.com/post/love-is-all-you-need (2018).

[71] Corral A (2021). Tail of the distribution of fatalities in epidemics. Phys. Rev. E.

