# Supplementary material for: Lognormals, power laws and double power laws in the distribution of frequencies of harmonic codewords from classical music
Source: Sci Rep. 2022 Feb 16;12:2615. doi: 10.1038/s41598-022-06137-3 (PMC8850585; doi:10.1038/s41598-022-06137-3)
Supplement: Supplementary file 1 — Supplementary Information. [file 41598_2022_6137_MOESM1_ESM.pdf]

Supplementary Information: Lognormals, Power Laws and Double Power Laws in the Distribution of Frequencies of Harmonic Codewords from Classical Music

Marc Serra-Peralta,[1,2] Joan Serrà,[3] and Álvaro Corral[1,4,5]

[1] *Centre de Recerca Matemàtica, Edifici C, Campus Bellaterra, E-08193 Barcelona, Spain*

[2] *Departament de Física, Facultat de Ciències, Universitat Autònoma de Barcelona, E-08193 Barcelona, Spain*

[3] *Dolby Laboratories, E-08018 Barcelona, Spain*

[4] *Departament de Matemàtiques, Facultat de Ciències, Universitat Autònoma de Barcelona, E-08193 Barcelona, Spain*

[5] *Complexity Science Hub Vienna, Josefstädter Straße 39, 1080 Vienna, Austria*

DATA PROCESSING AND FITTING

Data Cleaning and Detection of Repeated Pieces

As mentioned before, very short pieces are removed, and for us this is given by a number of beats below or equal to 15. In order to identify files corresponding to repeated pieces, we check if two or more files yield the same values of L and V. As this is not enough to characterize a repetition, we do the same analysis for bigrams, trigrams, and quadrigrams (a bigram is a concatenation of two codewords, a trigram of three, etc.), and the pieces having the same values of L and V from unigrams (our standard codewords) to quadrigrams are visually inspected, and the repeated ones removed. The number of repetitions was found to be 212. The resulting final values of L and V for the global corpus (76 composers with 9327 pieces) are $L = 5,088,442$ and $V = 4085$.

Harmonic Codewords

The elementary entities of which each musical piece is composed are defined as in Ref. [1] (in a similar way as it was done before in Ref. [2]). A summary of the procedure to obtain them follows:

(i) Starting with a MIDI file, corresponding to the score of a piece, this is converted by means of the `midi2abc` program [3] into a standard readable text file, containing the time occurrence, duration, and pitch of each note.

(ii) All pitches are transformed into pitch classes, which removes the distinction between different octaves. This leads to 12 pitch classes, C, $C\#$, etc., up to $A\#$ and B.

(iii) The piece is divided into time intervals corresponding to beats, as the beat is the fundamental time scale in music. Silent time intervals at the beginning and at the end of the piece are removed.

(iv) For each time interval, the sum of the duration of all the notes of a given pitch class are assigned to one of the 12 components of a 12-dimensional vector (called chroma), with each component associated to a pitch class, $(C, C\#, \ldots G\#, A, A\#, B)$. Notes

that occupy more than one time interval are split proportionally between the intervals. The duration is measured in units of the time interval (i.e., in units of beats).

(v) The vectors are discretized, with components below a fixed threshold (0.1 by default) reset to zero and components above the threshold reassigned to one. These "discretized chromas" yield the harmonic codewords or types that constitute the harmonic building blocks of the musical pieces. Note that there are $2^{12} = 4096$ possible types. So, at this coarse-grained level, each piece is represented as a 12-dimensional time series of binary elements.

(vi) Each piece is transposed; major pieces to C major and minor pieces to A minor. This constitutes a shift of the pitches by a constant amount (or a rescaling of the wavelengths, in physical terms), in order that the tonic note becomes pitch class C (for major keys) or A (minor keys). Thus, pitch classes turn to represent in fact tonal functions. See Ref. [1], which also details the method used for the determination of the key.

(vii) All pieces corresponding to the same composer are aggregated. This is done at the level of the time series of (transposed) discretized chromas. The reason for aggregation is that many pieces are too short to provide a meaningful statistical analysis. The resulting aggregation yields 76 datasets, one for each composer. An additional dataset, further aggregating the 76 individual datasets, is considered as representative of "classical music" as a whole. We refer to it as the global dataset.

Notice that step vi (transposition) would be irrelevant if we calculated the distribution of codeword counts for individual pieces, but it becomes very important as we aggregate the pieces, because they usually come in different keys (transposition makes the key to be the same, for pieces in major and minor keys, separately).

Fitting Method

For each dataset, the absolute frequencies n of each of the V types provides the values of the random variable for which the statistical analysis is performed (other approaches use the so-called rank as the random variable [4], but there are good arguments in favor of

using n [5]). The set of values of n allow one to apply maximum-likelihood estimation (once a fitting distribution is provided), as well as the Kolmogorov-Smirnov goodness-of-fit test (the latter through the construction of the estimated cumulative distribution function). In principle, a fit is accepted (in the sense that it is not rejected) if the resulting $p-$value of the Kolmogorov-Smirnov test is greater than 0.20 (note that this is much stricter than the usual 0.05 significance level). $p-$values are calculated from 100 Monte-Carlo simulations of the distribution resulting from the fit.

Fits are not performed for all $n \geq 1$, but above the lower cut-off a, greater than one in practice. The reason is that it is nearly impossible that a simple probability distribution (with few parameters) fits well the smallest frequencies, for which the statistics is very high (which is a common characteristics of Zipfian systems [5]); we systematically obtain $p = 0$ if $a = 1$. Thus, fits are repeated for different values of the lower cut-off, which are swept uniformly in log-scale (with 20 values per order of magnitude). In the case of the truncated power-law, there is also an upper cut-off b in the definition of the distribution, and, in the same way, different values of the upper cut-off are analyzed.

Among all non-rejectable fits (all with $p > 0.20$, for different values of the cut-offs), the procedure we use selects the one that includes more types. If the resulting value of the lower cut-off a is larger than 32, the fit is rejected (despite $p > 0.20$), due to the fact that more than one and a half orders of magnitude in frequencies (from $n = 1$ to $10^{3/2} \simeq 32$) would be excluded from the fit. Thus, $p > 0.20$ and $a \leq 32$ is what we consider a "good fit". Note that when we quantify the span of the fitting range of a distribution we refer to the logarithmic span of the fitting range, n_{max}/a, with n_{max} the maximum value of n observed in the data. An important thing about the fitting method is that it is fully automatic and does not involve subjective judgements (so as to decide when the power-law regimes start). More details are given in Refs. [6, 7].

Model Selection

When more than one distribution fits the data, we need to decide which is the best fit, in order to select it as the one best explaining the data. For model comparison, it is important to correct for the different number of free parameters that each distribution may have (the more parameters, the better the fit, in principle, but the improvement has to be significant).

For that purpose, it is common to use the Akaike and the Bayesian information criteria, or a likelihood-ratio test [8].

The problem we deal with, however, has an extra ingredient that makes the situation more complicated: each distribution fits a different subset of the data, in general, given by different values of the lower cut-off a for each distribution (a_{pl}, a_{dpl} and a_{ln}); i.e., only values of the random variable fulfilling $n \geq a$ are fitted (the criteria mentioned in the previous paragraph compare fits acting over fixed data). To overcome this difficulty, we use the simple criterion of choosing the distribution that fits a larger number of data points (i.e., more types), which is equivalent to select the one with the smaller lower cut-off a, independently of the number of parameters. This is what defines for us the "best fit," and in this way, we give absolute priority to fitting the largest fraction of the dataset.

The reason for our choice can be justified as follows. If a distribution fits the data for $n \geq a$, then, the remainder $a - 1$ empirical points (outside the fit) can be considered to be fitted using the $a-1$ values of the empirical probabilities $f(n)$, from $f(1)$ to $f(a-1)$. Thus, if the difference in $a-$values between two distributions is relatively large, as it is the usual case, the difference in the number of "total parameters" (including the empirical probabilities below a) is large as well and the distribution with smaller a has a much smaller number of "total parameters." Note that, in any case, we are comparing distributions that already provide good fits ($p > 0.20$). Moreover, we find more natural to prefer a distribution given by a simple formula, than one containing empirical values of probabilities as free parameters. In the case of two distributions yielding the same minimum value of the lower cut-off (tie), the distribution with a smaller number of parameters is selected as the best fit (we only find this situation in 7 cases).

---

[1] M. Serra-Peralta, J. Serrà, and A. Corral. Heaps' law and vocabulary richness in the history of classical music harmony. *EPJ Data Sci.*, 10:40, 2021.

[2] J. Serrà, A. Corral, M. Boguñá, M. Haro, and J. Ll. Arcos. Measuring the evolution of contemporary western popular music. *Sci. Rep.*, 2:521, 2012.

[3] abcMIDI package. *http://abc.sourceforge.net/abcMIDI/original*, 2003.

[4] E. G. Altmann and M. Gerlach. Statistical laws in linguistics. In M. D. Esposti, E. G.

Altmann, and F. Pachet, editors, *Creativity and Universality in Language. Lecture Notes in Morphogenesis*. Springer, 2016.

[5] A. Corral, I. Serra, and R. Ferrer-i-Cancho. Distinct flavors of Zipf's law and its maximum likelihood fitting: Rank-size and size-distribution representations. *Phys. Rev. E*, 102:052113, 2020.

[6] A. Deluca and A. Corral. Fitting and goodness-of-fit test of non-truncated and truncated power-law distributions. *Acta Geophys.*, 61:1351–1394, 2013.

[7] A. Corral and A. González. Power law distributions in geoscience revisited. *Earth Space Sci.*, 6(5):673–697, 2019.

[8] A. Clauset, C. R. Shalizi, and M. E. J. Newman. Power-law distributions in empirical data. *SIAM Rev.*, 51:661–703, 2009.
